# Fecal steroids of breeding and non-breeding free-ranging black-tufted marmoset females

**DOI:** 10.1590/1984-3143-AR2024-0003

**Published:** 2024-11-18

**Authors:** Ita de Oliveira e Silva, Vanner Boere, Maria Bernardete Cordeiro de Sousa

**Affiliations:** 1 Universidade Federal do Sul da Bahia, Ilhéus, BA, Brasil; 2 Universidade Federal do Rio Grande do Norte, Natal, RN, Brasil

**Keywords:** Callithrix penicillata, hormones, marmosets, reproduction

## Abstract

The relationships between members of the groups include behaviors related to affiliation, dispute for dominant positions, parental care, and facing disputes for food and territory. All these activities are under hormone modulation and those of a steroidal nature are heavily involved. Despite this, only few data are available on steroid hormones in free-ranging marmosets of the *Callithrix* genus, which limits the understanding of the physiological functioning and modulation of the socio-sexual behavior by steroid hormones of this taxon. In this study, we characterized fecal concentrations of progesterone, estrogens, and glucocorticoids of six breeding and non-breeding females from two groups of free-ranging *Callithrix penicillata (É. Geoffroy, 1812).* The concentration of progesterone was significantly higher in females which gave birth, compared to non-breeding females. The levels of fecal estrogens and glucocorticoids did not differ between breeding and non-breeding females. The data are in agreement with the few studies on steroid values of wild and captive marmosets. This study shows the concentrations of progesterone and glucocorticoids in free-ranging *C. penicillata* for the first time, and it is the only study reporting the concentration of fecal estrogens in wild marmosets. Overall, the high levels of progesterone associated with pregnancy in free-ranging *C. penicillata* as well as levels of estrogens and glucocorticoids close to those reported for other species, suggest a conserved pattern of hormonal secretion between *Callithrix* species that have been studied in captivity.

## Introduction

In many animal species, the breeding females have a higher and faster steroidogenesis and steroid metabolism than non-breeding females, which results in different sex steroid concentrations for breeding females and non-breeding females (Noyola-Martínez et al., 2019). In some primates, dominant and subordinate females that live in captivity show significant differences in the concentrations of ovarian steroids (estradiol and progesterone), indicating, respectively, ovarian function or its inhibition (Mustoe, 2023). Researchers found the same patterns for progesterone in field studies of *C. jacchus*, a primate of the Callitrichidae family (Sousa et al., 2005; Albuquerque et al., 2001). As reproduction is a metabolic challenge that demands high availability of energy and has behavioral costs, it is expected that there will be also an increase in glucocorticoids which are related to stress, energy metabolism, and immunity (Sapolsky et al., 2000).

Marmosets respond to psychosocial or physical stressors such as the Old World primates. However, unlike other Old World Primate species, in physiological conditions, captive marmoset (e. g., *C. jacchus*) have high plasma glucocorticoid concentrations, but there are no pathological signs of hypercortisolemia (Abbott et al., 2003). Studies on captive marmosets have also found high plasma concentrations of progesterone, but without signs of uterine hyperplasia (Abbott et al., 2003). This is because steroids are weakly linked to plasma proteins. In these callitrichids, the presence of an FK-506 binding immunophilin inhibits the connection of steroids to plasma proteins, making this taxon of primates “resistant to steroids”. Despite this evidence from studies in captivity, in free-range marmosets, as far as we know, there is no data that elucidate whether glucocorticoids are high in reproductive females or non-reproductive females.

Breeding females of the Callitrichidae family are known for experiencing a high re-productive cost, which leads to socioecological adaptation and high levels of steroid hormones (Tardif et al., 1993). In groups of the *Callithrix* genus, reproduction appears to be limited to one breeding female in most captive studies (*Callithrix kuhli*, Smith and French, 1997); *C. jacchus*, Mustoe, 2023). Pheromonal and behavioral mechanisms, mainly performed by the reproductive female, inhibit the sexual cycle of other adult females, pushing the marmosets towards a monogamous mating system in captivity (Tardif et al., 2003).

The reproduction of marmosets seems more flexible in the wild, since monogamous as well as polygynous groups were recorded (Yamamoto et al., 2009). Some studies show that free ranging marmosets can have more than one breeding female in the same group (*C. jacchus*, Roda and Pontes, 1998; Arruda et al., 2005; Yamamoto et al., 2009); *Callithrix flaviceps*, Hilário and Ferrari, 2010). Additionally, for common marmosets, the hormonal profile for two pregnant females (mother and daughter) and that from a non-reproductive females were also characterized in wild (Albuquerque et al., 2001; Sousa et al., 2005). Breeding female has a high cost for regular twin pregnancies that last around 5.5 months, delivering offspring that are born with a high infant/mother weight ratio (up to 17%, (Tardif et al., 1993). A few weeks after parturition, breeding females can start a new gestation because they have fertile postpartum estrus, without the occurrence of prolactogenic anestrus (Tardif et al., 1993; Hilário and Ferrari, 2010). Added to this reproductive load, there is a high energy cost to breastfeeding two offspring for a period of two to three months during a new pregnancy (Dixson and Lunn, 1987; Tardif et al., 2001). Therefore, it is expected that hormones associated to reproduction and metabolic costs increase in breeding females when compared to non-breeding female marmosets.

Most studies on the steroidal endocrine concentrations have been carried out on *C*. *jacchus* in captivity, with some field studies that shed new light on marmoset socioecology (Albuquerque et al., 2001; Sousa et al., 2005). However, five other *Callithrix* species remain understudied with regard to a number of socioecological and hormonal aspects. It is not known whether the physiological concentrations of steroids are unique in each category of female (reproductive or non-reproductive), in each species of marmoset. However, the most recent findings on natural hybridization among various *Callithrix* species (Malukiewicz et al., 2015) suggest that there is little, if any, endocrine or reproductive barrier between species of this taxon.

These few and incipient findings, leads us to hypothesize that the steroid concentrations are similar between breeding females from different *Callithrix* species. For example, free-ranging hybrid marmosets of *C. penicillata* x *C. jacchus*, *C. penicillata* x *C. geoffroyi*, have been found in places as far away as the states of Pernambuco, Rio de Janeiro, Minas Gerais, and Espírito Santo (Malukiewicz et al., 2015; Malukiewicz, 2019; Vale et al., 2020). These hybrid marmosets are fertile and have bred in many of these locations (Tardif et al., 2001; Malukiewicz, 2019). Hybridization in many causes yet to be studied. Malukiewicz e collaborators (2015) suggests that erratic introduction, destruction of natural barriers and alteration of habitats facilitated the hybridization of *C. penicillata* with other species of marmosets. Additionally, the larger distribution area of *C. penicillata* may also facilitate areas of contact with other species of marmosets (Vale et al., 2020). However, hybridization would not be possible if the different species of marmosets did not have compatible hormonal and socio-sexual patterns.

Steroids hormones of captive female *C. jacchus* are well studied, despite highest values variation due to the estrus cycle (Kramer and Burns, 2019). No data are available for steroid hormone concentrations of free-ranging *C. penicillata* females. Studies on the endocrine profile of free-ranging marmosets can be a referential support for studies on physiology, socio-ecological aspects, management, and conservation of free-ranging marmosets (Tardif et al., 2001). Therefore, given some information about endocrine steroidal values, properly contextualized in the natural history of *C. penicillata*, it is an original contribution that may help to understand the adaptive role of steroids in the ultimate and proximal causes related to behavioral ecology and reproductive mechanisms in *C. penicillata*. In light of these gaps on studies of free-ranging marmoset steroids, the aim of this investigation is to provide, for the first time, the levels of progesterone, estradiol and cortisol in free-ranging *C. penicillata* females.

## Methods

The studied animals (*C. penicillata*) inhabited a region of mesophytic forest and dense savannah, in the Botanical Garden of Brasília (15°52’S and 47°50’W; 1056 m altitude), Brazil. The area has trails and narrow access roads, and is suitable for public visitation. The individuals observed belonged to two groups living in adjacent areas that were habituated to the presence of humans. Groups I and II were neighbors and inhabited areas with little overlap. Eventually there were intergroup direct contact that were characterized by agonistic displays or fights took place by adult individuals. It is suggestive that the area of use was defended by each group. During 22 months of study, Group I had a size that ranged from 15 to 19 individuals, where four adult females (named FDM, FCT, FBC and FBR) can be identified. Group II varied in size from 11 to 14 individuals, with three adult females named FDM2, FBL and FLD.

We avoided capturing individuals, to avoid stress, but the degree of habituation al-lowed marmosets to researcher approach at distances of two meters or less. This condition allowed us to look at the size of the abdomen, breasts and vulva. Therefore, we are very confident in stating the advanced pregnancy in both, groups I and II. The age range estimate was based on data described by (Tardif et al., 2003), for the species *C. jacchus*. Based on dentition characteristics, FDM appeared to be the oldest female in Group I. For the other females, it was not possible to distinguish characteristics to differentiate them by age, except that they appeared younger than FMD from group I.

The collection of feces and the hormonal analyzes of the present study were opportunistic, due to a semi-naturalistic experiment on access to food resources of female marmosets. To collect fecal samples, we used a platform consisted of a wooden structure with a metallic grid floor measuring 120 cm (length) x 60 cm (width) x 20 cm (depth), held by a 110-cm-high support. Ten cube trays, with 10 holes each, were placed under the grid floor. Marmosets easily eat bananas, which seem highly appetizing. Therefore, 20 banana slices, approximately 1.0 cm thick each, were placed in the cube trays in the center of the platform. During each feces collection time, the individuals quickly approached and descended on the platform to pick up and eat the banana slices. On some of these occasions, the marmosets defecated on the grate, with the feces dripping onto the small cubes from which they were collected. An observer recorded which individuals had defecated. After each observation session, the platform and cubes were sanitized. This procedure lasted 30 minutes, and was randomly repeated for two days every month, over a period of 18 months.

Fecal samples were identified, placed in vials, and stored in a freezer (-20°C) to later be submitted to laboratory procedures. To avoid interference from physiological circadian variations, all material was collected between 6 am and 10 am. Hormonal analyses of progesterone, estrogen, and cortisol were performed by ELISA immunoassays in the Laboratory of Hormonal Measurement at the Federal University of Rio Grande do Norte, Brazil, following the protocol described by Ziegler and Sousa (2002). Before performing the enzyme immunoassays, hormonal steroids were extracted from the feces by hydrolysis and solvolysis following the protocol describe by Ziegler and Sousa (2002).

The statistical analysis of the data was performed using SPSS 20.0 for Windows. The estrogen and glucocorticoid concentrations were not normally distributed, therefore we carried out the non-parametric Mann-Whitney test to compare all fecal steroids concentrations between reproductive females and non-reproductive females (Zar, 1999). All distributions were assumed two-tailed and the significance level was less than 5%.

### Ethical considerations

This study, like others before 2008 (e. g., Roda and Pontes, 1998; Albuquerque et al., 2001; Arruda et al., 2005; Boere et al., 2005; Sousa et al., 2005; Yamamoto et al., 2009) did not require formal licensing from an Animal Ethics Committee. In Brazil, there were no laws regulating the behavioral observation and collection of biological material from wild primates until 2008. Federal Law Number 11794/2008 regulated the procedures for the scientific use of animals, requiring the National Council for the Control of Animal Experimentation, to supervise and monitor registered Research Institutes, which use animals in their experimental procedures with the support of the Ethics Committee on the Use of Animals – CEUAs (Guimarães et al., 2016). The present study was carried out between the years 2006 and 2007, precluding licenses to observe wild marmosets. Notwithstanding the unnecessary formal request for a license, all procedures followed ethical standards and no harmful events for marmosets occurred during the study (Tardif et al., 2006). The Brazilian Institute for the Environment and Renewable Resources (IBAMA) and the Botanical Garden of Brasilia, formally allowed the research to be conducted. The collection of material was monitored by a biologist and a veterinarian. No marmosets were injured or died accidentally due to observations or material collection during the study.

## Results

**Figure 1:**
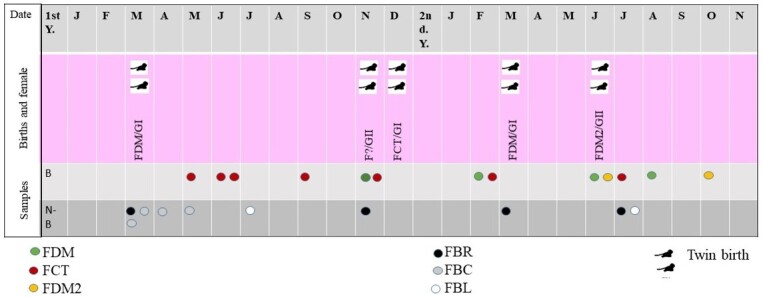
Sequence of offspring births during the two years of observations and fecal samples per animal. FDM, FDM2, FCT are the identification of the breeding females. GI, group I; GII, group II. The marmosets sampled were of the Jardim Botânico de Brasília, Federal District, Brazil. The study was carried out between January 2006 (1^st^. Y.) and October 2007 (2^nd^.Y.). Each year is from January (J) to December (D), but 2^nd^ Y is only until November (N). Each silhouette of two marmosets signifies the sighting of two infants by the observer. Each circle represents a sample of feces and each color distinguishes the different females in the study. Samples are the month of fecal collection: B (breeding females), N-B (Non-breeding females).

The female from Group I known as FDM gave birth to twins in March of the first year and in March of the second year of observations (Figure 1). The female from Group I known as FCT gave birth to twins in December of the first year of observations. In Group II, only one birth was observed for female FDM2, in June of the second year of observations. At the beginning of the second year observations, there were four infants approximately two months old, suggesting that there were births in Group II. It was not identified from which females these four offspring originated (Figure 1). The infants whose mother could not be identified suddenly appeared in group II, during the month of November of the first year. The infants appeared to be two months old. We strongly suspect that these two infants that “appeared” in group II were offspring of female FDM2. Due to the birth identified mother and his offspring, FDM, FCT, and FDM2 were considered breeding females.

In Group I, four fecal samples were collected from FDM and FBR, four samples from FBC and seven samples were collected from FCT. In Group II, only two samples were collected from FDM2 and two samples from FBL. Samples were not obtained from FLD because she did not defecate on the platform. The results are summarized in Table 1.

**Table 1:** Mean and standard error (Mean±SE) of fecal levels of progesterone, estrogens, and glucocorticoids for each free ranging female *Callithrix penicillata*. The marmosets sampled were of the Jardim Botânico de Brasília, Federal District, Brazil. The study was carried out between January 2006 and October 2007.

**Group/Female**	**Reproductive status**	**Progesterone (ng/g)**	**Estrogens (ng/g)**	**Glucocorticoids (ng/g)**
		**Mean±SE**	**Mean±SE**	**Mean±SE**
Group I				
FDM	B	398.59±89.20	51.93±70.29	112.06±21.28
FCT	B	524.43±91.06	32.78±15.58	140.14±38.75
FBC	NB	283.93±73.87	348.70±682.75	63,82±38.41
FBR	NB	263.50±103.69	14.75±7.93	71.19±15.61
Group II				
FDM2	B	777.48±381.45	130.03±123.31	2277.99±2014.34
FBL	NB	170.62±1.84	4.86±1.77	130.81±35.19

B: breeding female (n=3); NB: non-breeding female (n=3). Number of samples collected: FDM, 4; FCT, 7; FDM2, 2; FBR, 4; FBC, 4; and FBL, 2.

**Figure 2:**
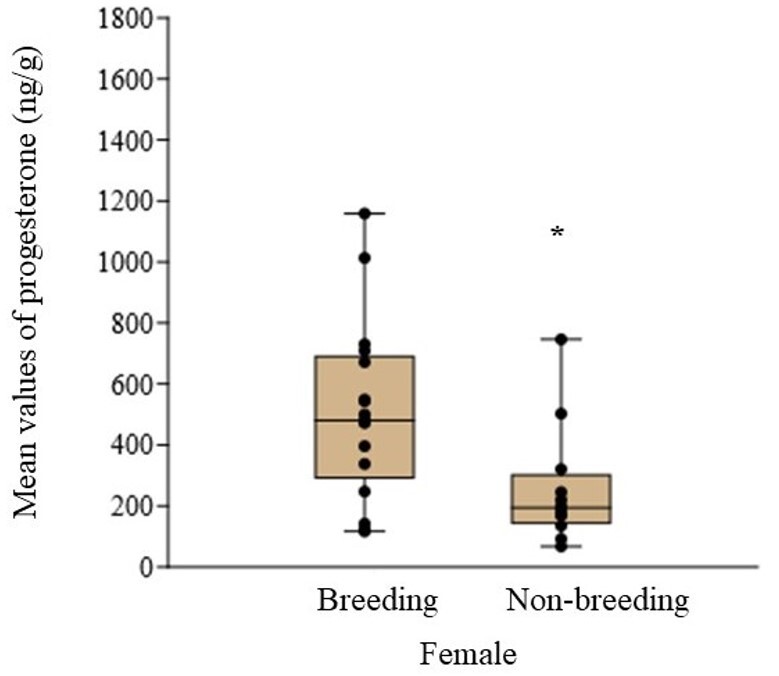
Mean values of progesterone levels (ng/g feces) for breeding (n=3) and non-breeding (n=3) females of free ranging *Callithrix penicillata*. The black line across the box is the median (Breeding females=480.46; Non-breeding females= 193.93). The black dots are each individual's fecal progesterone values. Mann-Whitney test, d. f.= 18, Z=2,3247, P = 0.02*.

We found significantly higher concentrations of progesterone for breeding females compared to non-breeding females (Figure 2; Mann-Whitney test, d.f.= 18, z=2,3247, P = 0.02). We did not find statistical differences between breeding females and non-breeding females, for estrogen (Mann-Whitney, d.f.= 18, Z = 0.44, P = 0.66) or glucocorticoid concentrations (Mann-Whitney, d.f.= 18, Z = 1.30, P = 0.19). The intra-assay coefficients of variation were 10.62% + 2.62% for estrogens, 7.46% + 5.5% for progesterone, and 3.61% + 2.43% for glucocorticoids, respectively.

## Discussion

Higher levels of progesterone were observed in breeding females, but the fecal concentrations of estrogens and glucocorticoids were similar, regardless of the females' reproductive status. The range of variation of fecal steroids for free-ranging *C*. *penicillata* females is similar to that observed in females of other species of the genus *Callithriix*, both in captivity and in a wild environment.

Progesterone and its metabolites are the principal hormones associated with pregnancy, and are widely used to indicate reproductive condition in females of *C. jacchus* (Sousa et al., 2005; Alencar et al., 2006). In the wild and in captivity, breeding *C. jacchus* females have higher levels of plasma (Alencar et al., 2006), as well as urinary (Kramer and Burns, 2019) and fecal progesterone (Albuquerque et al., 2001; Sousa et al., 2005). There is a lack of data in the scientific literature on progesterone concentrations in free-ranging females from other species of the genus *Callithrix*. In captivity, females of *C. kuhli* showed higher levels of urinary progesterone during pregnancy and in the phase just after the luteinizing hormone peak (French et al., 1996). Mustoe et al. (2012) studied the estrous cycle of three *C. geoffroyi* females, in gestational and non-gestational phases, and concluded that there was an increase in urine progesterone during pregnancy. The significant differences in the concentration of fecal progesterone from reproductive females of both, group I and group II, compared to non-breeding females, is according to progesterone levels observed in other *Callithrix* species (Sousa and Ziegler, 1998; Ziegler and Sousa, 2002).

The increase in progesterone and estradiol in humans, catarrhines and platyrrhine primates related to sexual cycle is well documented in the scientific literature. In *C. jacchus* living in captivity, progesterone increase is followed by estradiol sustained elevation during the luteal phase (Ziegler and Sousa, 2002). In the present study, breeding and non-breeding free-ranging *C. penicillata* females had variable estrogen concentrations, but there was no substantial difference between them. This is likely occurring because estrogen levels rise and fall during a normal sexual cycle and sampling has not been performed regularly due to the limitations of our sample collecting schedule. Regarding these fluctuations in estrogen levels, this is in agreement with some studies on adult breeding and non-breeding *C. jacchus* females in captivity, which show no differences in the concentration of fecal, urinary, or plasma estrogens (Smith and French, 1997). On the other hand, as progesterone show a very high production pattern throughout the entire period of pregnancy in primates, including *C. jacchus* (Sousa and Ziegler, 1998), the difference was detected when this steroid from reproductive and non-reproductive females was compared.

In captive *C. kuhli* females, there is great variation in estrogens concentrations during the estrous cycle (French et al., 1996), both for breeding and non-breeding females. In other species of the genus *Callithrix*, such as *C. geoffroyi*, *C. aurita*, and *C. flaviceps*, there are no studies on estrogen concentrations for either free-ranging or captive females. In studies on *C. jacchus* and in the current study on free-ranging *C. penicillata*, there is wide variation in estrogen levels, attributed to the cyclical hormonal alterations of the estrous cycle, both for breeding and non-breeding females. Although most studies only observed one breeding female, some non-breeding females also have a regular estrous cycle (Sousa et al., 2005). In some cases, estrogen concentrations were low, suggesting the lack of an estrous cycle in non-breeding females, which may be dependent on socio-sexual context (Alencar et al., 2006).

The basal glucocorticoid levels in *Callithrix* genus are higher than all Catarrhines species studied so far (Abbott et al., 2003). Studies on basal concentrations of glucocorticoids in free-ranging *C. penicillata* were not found in the literature review. Therefore, the data in this study was compared against the only study of free-ranging *C. jacchus*, where cortisol levels are high and not differentiated between breeding and non-breeding females in three groups (Sousa et al., 2005). Considering data from studies on captive *C. penicillata*, these results are according that the concentration of glucocorticoids is high and does not differ between breeding and non-breeding females (Smith et al., 2011). These results are also in agreement with studies on other species of the genus *Callithrix* in captivity (*C. kuhli*, Tardif et al., 1993; *C. jacchus*, Ash et al., 2018; *C. geoffroyi*, Smith et al., 2011), in which the concentration of glucocorticoids was not influenced by the reproductive condition.

Some studies on captive individuals have linked the increase in glucocorticoids to social challenges, but not to the reproductive condition (Tardif et al., 1993). Other stressful situations seem to strongly provoke an increase in glucocorticoid, such as unpredictability and resource control (Pereira et al., 2020), exposure to models of predators (Cross and Rogers, 2006), forced withdrawal from the social group, and proximity to unfamiliar individuals (Smith et al., 2011). The females observed in this study lived in their own social groups that have social buffering mechanisms, such as behaviors (contact, grooming, and copulations) that reinforce family bonding and lower levels of glucocorticoids (Sanchez et al., 2015; Pereira et al., 2020). Thus, the results of this study indicate that glucocorticoids are constitutively high, but independent of reproductive status in free-ranging *C. penicillata* females.

### Study limitations

Our study has many limitations. The main limitation is the small sample size to allow tracing the entire estrous cycle and pregnancy events of marmosets. To determine estrous cycle and steroid profile, it is necessary a day-to-day collection during many months (Kramer and Burns, 2019). Studies on primates in a natural environment have complex logistics and high costs, requiring parsimony in data collection. Additionally, even using our collection apparatus, the females rarely defecated on the platform where the collection cubes were. The present study did not aim to describe a complete hormonal changes of the estrous cycle of free-ranging female *C. penicillata*. Therefore, the data in this study did not cover the estrous cycle phases, because the goal was to determine steroid values from feces randomly, opportunistically, and non-invasively collected samples throughout the year.

Fresh feces are good material for hormonal analysis, but the concentrations of glucocorticoids found may be related to acute stressful events that occurred a few hours or the day before collection. Thus, glucocorticoids are not necessarily a chronic physiological or pathological condition of the individual.

It is understandable and accepted that the hormonal data collected from small samples of primates are important in composing the endocrine and social mosaic of many species, as can be observed in other studies (Sousa et al., 2005). Our study is a rare window, as insightful as possible, into hormonal aspects of reproduction in wild marmosets. We believe that the findings allow insights and new perspectives on endocrine and socio-sexual aspects of marmosets.

## Conclusions

In this study, for the first time, the levels of fecal steroids excreted by free-ranging female *C. penicillata* is evidenced. The fecal concentration of progesterone was higher in breeding female marmosets, probably due to their high secretion pattern over the five months of gestation, but estrogens and glucocorticoids did not differ significantly between breeding and non-breeding females. Based on these results, it can be suggested that the steroid values of *C. penicillata* are similar to those of other studied species of the genus *Callithrix*, regardless of whether they are captivity or free-ranging individuals. Therefore, it is suggested that a conserved pattern of secretion of steroids in *Callithrix* genus.
